# Integrative QTL mapping and selection signatures in Groningen White Headed cattle inferred from whole-genome sequences

**DOI:** 10.1371/journal.pone.0276309

**Published:** 2022-10-26

**Authors:** Rayner Gonzalez-Prendes, Catarina Ginja, Juha Kantanen, Nasser Ghanem, Donald R. Kugonza, Mahlako L. Makgahlela, Martien A. M. Groenen, Richard P. M. A. Crooijmans

**Affiliations:** 1 Animal Breeding and Genomics, Wageningen University & Research, Wageningen, The Netherlands; 2 BIOPOLIS/CIBIO/ InBIO, Research Center in Biodiversity and Genetic Resources, University of Porto, Vairão, Portugal; 3 Natural Resources Institute Finland, Jokioinen, Finland; 4 Animal Production Department, Faculty of Agriculture, Cairo University, Giza, Egypt; 5 Department of Agricultural Production, College of Agricultural and Environmental Sciences, Makerere University, Kampala, Uganda; 6 Agricultural Research Council-Animal Production Institute, Irene, South Africa; 7 Department of Animal, Wildlife and Grassland Sciences, University of the Free State, Bloemfontein, South Africa; University of Iceland, ICELAND

## Abstract

Here, we aimed to identify and characterize genomic regions that differ between Groningen White Headed (GWH) breed and other cattle, and in particular to identify candidate genes associated with coat color and/or eye-protective phenotypes. Firstly, whole genome sequences of 170 animals from eight breeds were used to evaluate the genetic structure of the GWH in relation to other cattle breeds by carrying out principal components and model-based clustering analyses. Secondly, the candidate genomic regions were identified by integrating the findings from: a) a genome-wide association study using GWH, other white headed breeds (Hereford and Simmental), and breeds with a non-white headed phenotype (Dutch Friesian, Deep Red, Meuse-Rhine-Yssel, Dutch Belted, and Holstein Friesian); b) scans for specific signatures of selection in GWH cattle by comparison with four other Dutch traditional breeds (Dutch Friesian, Deep Red, Meuse-Rhine-Yssel and Dutch Belted) and the commercial Holstein Friesian; and c) detection of candidate genes identified via these approaches. The alignment of the filtered reads to the reference genome (ARS-UCD1.2) resulted in a mean depth of coverage of 8.7X. After variant calling, the lowest number of breed-specific variants was detected in Holstein Friesian (148,213), and the largest in Deep Red (558,909). By integrating the results, we identified five genomic regions under selection on BTA4 (70.2–71.3 Mb), BTA5 (10.0–19.7 Mb), BTA20 (10.0–19.9 and 20.0–22.7 Mb), and BTA25 (0.5–9.2 Mb). These regions contain positional and functional candidate genes associated with retinal degeneration (e.g., *CWC27* and *CLUAP1*), ultraviole*t* protection (e.g., *ERCC8*), and pigmentation (e.g. *PDE4D*) which are probably associated with the GWH specific pigmentation and/or eye-protective phenotypes, e.g. Ambilateral Circumocular Pigmentation (ACOP). Our results will assist in characterizing the molecular basis of GWH phenotypes and the biological implications of its adaptation.

## Introduction

Traditional native breeds are an important source of genetic variability adapted to local environments. They might harbor genetic variants unique to the breed due to ecosystem adaptation and, e.g. provide resistance to local diseases and/or extreme climatic conditions. Detailed analyses of the genomic structure of those native breeds can contribute to improving the knowledge about breed formation, and identify genes and variants with a significant impact on the adaptation processes that shaped animal phenotypes [1–4]. This information can be used to set up optimum breeding programs for the management of livestock genomic resources.

The skin and coat color variation in livestock breeds are important traits that impact the adaptation of breeds to the environment [5–8]. In the past years, numerous research projects, such as genome-wide association studies (GWAS) [9–11] and whole-genome selective sweeps identification [3, 12] have been performed to pinpoint candidate genomic regions with significant effects on skin and coat color variation [6, 9, 10, 13–15]. The combination of several sources of information can improve the power of candidate gene identification by reducing the number of QTLs and their intervals, as well as providing additional insights into the studied biological processes [16, 17].

The Groningen White Headed (GWH) breed, originated from the Groningen province of the Netherlands, is a dual-purpose cattle known for its longevity, minimal veterinary costs, and high fertility rate [18]. The first GWH animal was registered in the herd book in 1875, and in 1999, the breed was considered to be endangered with approximately 830 purebreed animals [19]. Recent interest in functional traits such as fertility or resistance may open up new opportunities for the expansion of this breed [18]. GWH animals are easily distinguished by their phenotype, that is, solid black or red coat color, white face, and colored areas around the eyes [18, 19].

In cattle, Ambilateral Circumocular Pigmentation (ACOP) can be distinguished by a white face and colored areas around the eyes in breeds such as the GWH [19] and Fleckvieh [9]. The presence of this phenotype can reduce the susceptibility to eye lesions [20]. It is well-known that non-pigmented animals have a higher incidence of eye lesions than animals with eye margin pigmentation [9, 21]. A plausible explanation for this is that cattle with a non-pigmented eye margin are exposed to more ultraviole*t* (UV) radiation in this region [9], which would be more intense and harmful in the tropical areas [22].

The molecular genetic background of GWH breed has not been extensively studied [23]. Therefore, the goal of this study was to gain further knowledge on the genomic basis of the GWH breed by analyzing whole-genome resequencing data to identify and characterize genomic regions that differ between GWH and other cattle breeds, and in particular to identify candidate genes associated with coat color and/or eye protective phenotypes. We studied the population structure of five Dutch traditional breeds, to evaluate the genetic distinction of the GWH, using two approaches, which are, a model-based clustering admixture analysis and a principal component study (PCA). Additionally, we implemented an integrative approach, to reduce the number of false positive candidate genomic regions, taking into account the findings from: a) a genome-wide association study using GWH with ACOP, breeds without the white head phenotype (Holstein Friesian, Dutch Friesian, Deep Red, Meuse-Rhine-Yssel and Dutch Belted) and other white headed breeds (Simmental and Hereford); b) scans for candidate selective sweeps in GWH cattle compared to those of four other traditional Dutch breeds (Dutch Friesian, Deep Red, Meuse-Rhine-Yssel, Dutch Belted), and the transboundary Holstein Friesian; c) identification of runs of homozygosity (ROH) in the GWH breed to reduce the number of false positive candidate selective sweeps, and d) identification of functional candidate genes in the genomic regions commonly detected by GWAS, selective sweeps and ROH hostpots.

## Materials and methods

### Ethics statement

This study was conducted following the animal experimentation policy of Wageningen University & Research. The cattle blood samples were collected by a veterinarian during yearly routine health inspections with written informed consent by the owners. Therefore, no Ethics Committee approval for animal care was needed for this research.

#### Animals

We used 170 animals from eight breeds. We first sampled 120 unrelated animals as part of the LEAP-Agri project OPTIBOV (https://www.optibov.com/) and in collaboration with the respective breed associations, including 5 Holstein Friesian; 21 GWH; 23 Meuse-Rhine-Yssel; 23 Dutch Belted; 24 Dutch Friesian; and 24 Deep Red. In total, 92 cows and 28 bulls were included in this study (for more details see **S1 Table**). Secondly, white headed animals with no ACOP were retrieved from two more breeds (25 Simmental and 25 Hereford) included in the 1000 Bull Genomes Project (Run9 version) [24, 25]. These 50 animals with completely white heads (lacking ACOP) were used only for the genome-wide association analysis to contrast against the GWH breed, which exhibits ACOP.

#### DNA sample preparation and sequencing

The GENTRA Blood kit (Qiagen N.V.) was used for the isolation of genomic DNA from EDTA blood samples. The quantification and quality of the obtained DNA were assessed using the Qubit fluorometer (Qiagen N.V.). DNA was paired-end sequenced (read length of 150 base pair) as single-indexed genomic libraries using the Illumina Novaseq6000 (Illumina Inc., USA). Finally, raw reads were preprocessed by trimming the adapter sequences and removing the reads with 50% of low-quality nucleotides and fewer than 36 base pairs in length with fastp v0.23.1 [26].

#### Short read alignment, mapping, variant detection, and filtering

The mem option from BWA v0.7.17-r1188 [27] was used to map the cleaned reads to the bovine reference genome (assembly version ARS-UCD1.2) [28]. Aligned reads from each animal were stored in binary BAM files using SAM tools v0.1.19 [29]. Freebayes software [30] was used for population-based variant calling with default parameters except for: -min-alternate-count = 3, -haplotype-length = 0, -ploidy = 2, -min-alternate-fraction = 0.2, and -min-base-quality = 30. Variants with a phred-scaled probability < 20 and a depth of coverage by sample <5 were removed using the Bcftools v1.9 [31] software.

#### Population structure assessment with principal component analysis and individual ancestry estimation

We used PC analysis to assess the population structure of the Dutch cattle breeds. This analysis was conducted using the variance-standardized relationship matrix [32] with PLINK v1.9 [32]. We considered only autosomal and biallelic variants with an r^2^ < 0.5 between variants within a window of 50 SNPs and with a genotyping rate > 0.95. The results from the PCA were visualized using the R package ggplot2 v3.3.5 [33].

Individual ancestry was evaluated by a model-based clustering method with the ADMIXTURE software v1.23 [34]. This method used the allele frequencies and the proportions of the ancestral populations in each sample to model the probability of the observed genotypes [34]. In the model, the K-value (optimal number of clusters) was estimated as the one with the lowest cross-validation error (CV) [34]. The ADMIXTURE algorithm was performed using values of *K* ranging between 2 and 6. The analysis was performed with a total of 120 unrelated animals from Dutch breeds and included 1,354,139 autosomal variants with a r^2^ < 0.5 within windows of 50 variants over the genome and a minor allele frequency (MAF) > 0.05.

#### Genome-wide association study

A genome-wide association study was used to identify and characterize genome regions that differ between GWH and other breeds to find out candidate genes funtionally related with pigmentation and/or the eye protective phenotypes, e.g. ACOP. We used a mixed-model approach developed by Zhou and Stephens [35] in the Genome-wide Efficient Mixed-Model Association v0.98.1 [35] program. The mixed-model approach accounted for the population structure by including in the random effect the covariance structure from the genomic kinship matrix. In a first step, the association analysis was performed between GWH and non-white headed Dutch breeds (5 Holstein Friesian; 24 Dutch Friesian; 23 Meuse-Rhine-Yssel; 23 Dutch Belted; and 24 Deep Red). A total of 14,285,317 autosomal variants with a MAF > 0.05 were used to evaluate the relationship between each variant and the GWH breed phenotypes:

y=Wα+xδ+u+ε

where ***y*** was the binary phenotype, one for the GWH individuals with ACOP and zero for Dutch Belted, Deep Red, Meuse-Rhine-Yssel, Dutch Friesian, and Holstein Friesian; **W** the matrix of incidence for the fixed effects; **α** the intercept vector of ones; **x** contains the vector with SNP genotypes by sample; **δ** represents the marker effect size; **u** contains a vector with the random genetic effects that follow a n-dimensional multivariate normal distribution with **u** ∼ MVN_n_ (**0**, λ *τ*^− 1^ **K**) for n individuals and being **λ** the ratio from two components of variance, *τ*^− 1^ is the variance of the residual error, and **K** the kinship matrix derived from the genotypes from each sample; **ε**  ∼ MVN_n_ (**0**, *τ*^− 1^ I_n_) the vector containing the errors, with I representing the identity matrix. The nominal *p-values* from the association study were corrected using the false discovery rate (FDR) approach implemented in the R function p.adjust [36] and Benjamini & Hochberg [37] method. We considered those variants with a *q-value* (from the FDR test) lower than 0.001 as significantly associated. Here, a QTL and the co-localization between QTLs and significant selective sweeps were defined following the method reported by Gonzalez-Prendes et al. [38]. In brief, we considered only genomic regions with more than two significantly associated variants as candidate QTL. The co-localization between QTLs or between QTLs and selective sweeps was considered if the genomic regions overlapped by at least one base pair.

In a second step, variants from two additional breeds (25 animals from the Simmental breed and 25 from Hereford) with white heads and no ACOP were retrieved from the 1000 Bull Genomes Project (Run9 version) [24, 25] to perform the GWAS between these and GWH. We decided to keep the analysis with those two transboundary breeds separated from the remaining five Dutch breeds because we used different approaches to detect variants from whole genome resequencing data and we did not want to lose informative variants segregating in the populations at low frequency for subsequent analyses. The Simmental and Hereford sequence data, with a mean depth of coverage of 11.68 X (between 1.84 and 44.17) [24, 25], were merged with the data obtained from the 120 animals in our study, including 21 GWH individuals using PLINK v1.9 [32] with default parameters. The association study was performed with a total of 9,655,666 variants with a genotype call rate above 0.9, a MAF higher than 0.05 and using the model described above.

### Identification and annotation of selective sweeps

The variants identified in each sample were used to explore the presence of genomic regions under selection in each breed with two complementary methods. First, Sweep Detector (SweeD) v3.0 [39] software, was applied using a composite likelihood ratio test to find candidate selective sweeps across the genome based on Site Frequency Spectrum patterns of variations [40]. We defined a window size of 5 kb across the genome to calculate the Site Frequency Spectrum patterns, and the outlier regions falling within the top 1% of the composite likelihood-ratio test distribution were selected as significant regions. Second, a complementary approach based on linkage disequilibrium implemented in OmegaPlus v3.0.3 [41] was applied. Here, the *ω*-*statistic* is calculated based on patterns of linkage disequilibrium close to a recently fixed mutation. A high value of *ω*-*statistic* at a specific genomic location can indicate a genomic region under selection. In this method, we used the same window size of 5 kb bins across the genome and outlier regions with the highest values (top 1%) of *ω*-*statistic* were considered significant. Finally, only candidate selective sweeps within the 1% of the highest scores obtained by both methods were annotated using Ensembl 101 [42] database and used for subsequent analyses.

#### Runs of homozygosity identification in the GWH breed

The detection of ROH in the GWH breed was implemented with detectRUNS v0.9.6 [43] program. This analysis was used as a complementary method to confirm and reduce the number of candidate genomic regions that co-localize between the GWAS signals and selective sweeps. Genomic regions with ROH hotspots were selected to control the number of false positive candidate selective sweeps and GWAS signals by selecting only genomic regions that co-localize between them. A sliding window-based method was applied to detect regions with at least 15 variants in a run with 250 kb as the minimum length and a maximum distance between consecutive variants of one Mb. Additionally, we considered one variant per 10 kb as the lower density limit and only one missing or heterozygous variant per run. Potential ROH hotspots were identified by selecting only genomic regions containing the most frequent (top 1%) variants in a run in the GWH population [44–46].

## Results and discussion

After the mapping of the Dutch cattle breeds and Holstein Friesian short read sequences to the bovine reference genome (assembly ARS-UCD1.2), the depth of coverage across samples, in average, was 8.7X ranging from 7X to 13X (**S1 Table**). The number of variants per breed, biallelic variants and variants that are specific to each breed are shown in **Table 1**. The overall number of annotated variants was 21,313,663, and the number of SNPs per animal (between 6 and 7 million, **S1 Table**) and per breed (between 13 and 17 million, **Table 1**) are within the range of that obtained in other studies on *B*. *taurus* [47–53]. The breed with the highest number of breed-specific variants was Deep Red (558,909), whereas the Holstein Friesian showed the lowest number (148,213). The low number of specific variants detected in Holstein Friesian compared with the remaining breeds in this study is most likely because of the small effective population size associated with a strong artificial selection pressure [54]. However, as the number of samples (n = 5) for Holstein Friesian is low, specific variants with low frequency may be underestimated and the results must be taken with caution. Functional annotation analysis revealed that the detected variants mapped to intronic (46.18%) or intergenic (42.61%) regions. Only, 1.1% (389,472 variants) mapped to exonic regions, of which 146,057 were missense and 212,473 were synonymous variants (**S2 Table**).

**Table 1:** Number of variants by breeds and breed-specific variants detected in 6 cattle populations.

Breeds	Mean genome coverage	Number of variants	Number of biallelic variants	Number of biallelic breed-specific variants
Holstein Friesian	9.20	13,218,695	12,376,751	148,213
Meuse-Rhine-Yssel	9.78	16,642,547	15,751,146	304,064
Groningen White Headed	8.19	15,554,352	14,675,130	368,389
Dutch Frisian	8.46	16,025,075	15,139,002	374,333
Dutch Belted	8.30	16,463,618	15,574,124	475,279
Deep Red	8.75	17,804,474	16,906,802	558,909
**Mean**	8.78	15,951,460	15,070,493	371,531

### Genetic differentiation of the GWH breed

**Fig 1:**
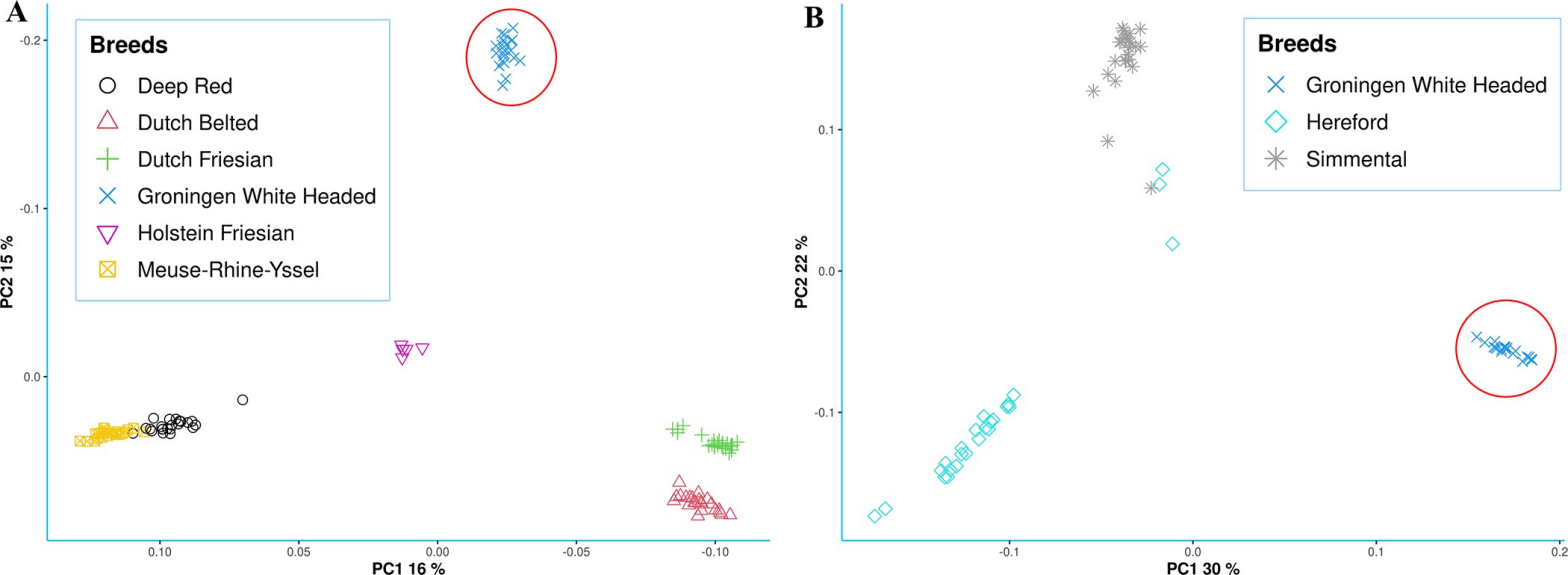
Principal component analysis (A) 120 samples from six breeds (Holstein Friesian, Dutch Friesian, Dutch Belted, Deep Red, Meuse-Rhine-Yssel and GWH); (B) The 70 animals from the three populations, GWH breed plus two white headed breeds (Hereford and Simmental) used for the GWAS in the second step. Individuals from the GWH breed (red circle) are distantly positioned from all other breeds in both plots. The % symbol indicates the percentage of the explained variance for the first and second components calculated from the eigenvalues.

**Fig 2:**
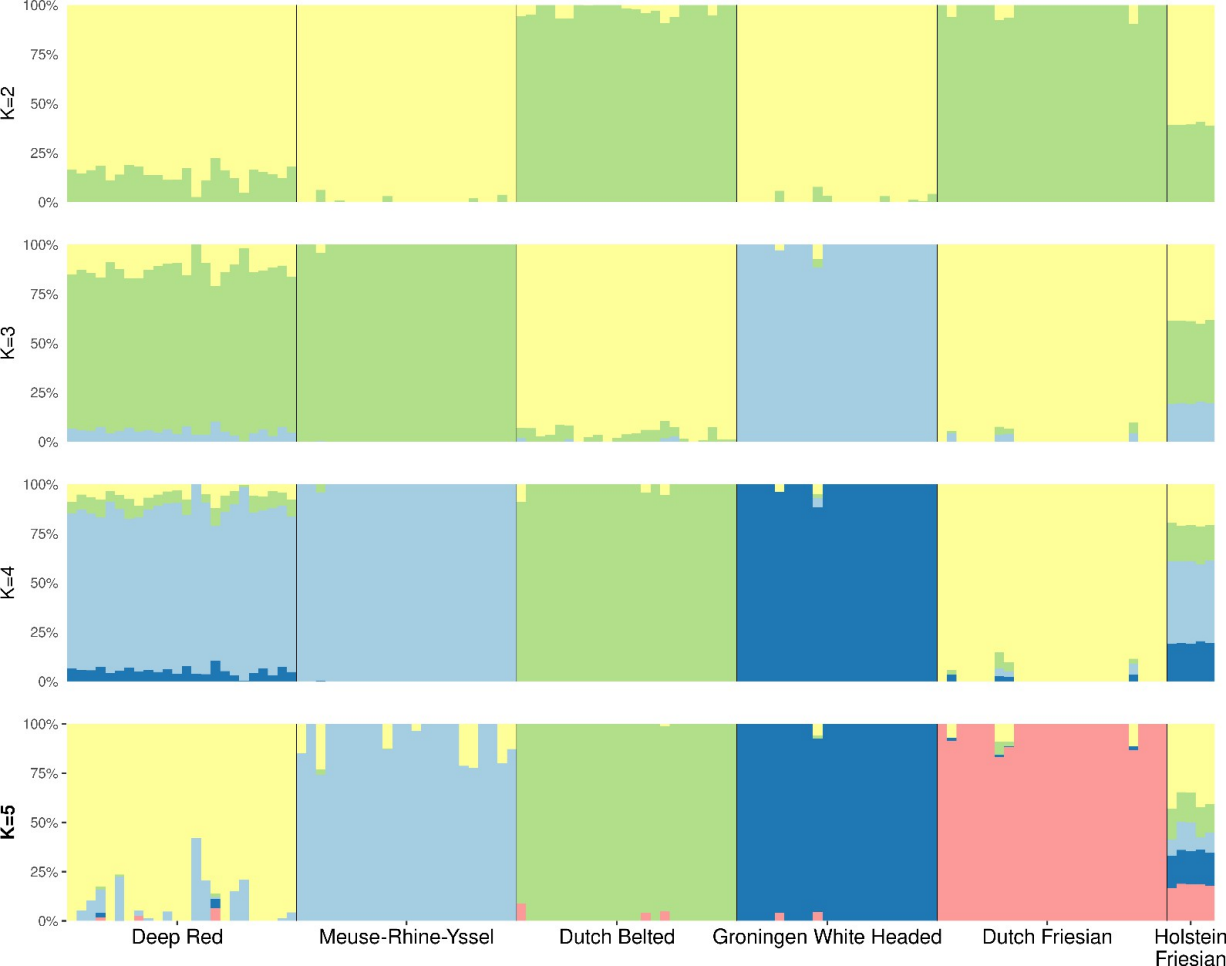
Population structure plot determined by the model-based clustering analysis of ADMIXTURE. Samples are represented by stacked columns of the 2 to 5 K-proportions and the number of clusters with the lowest cross validation error (CV = 0.54) was obtained for *K* = 5.

The genetic relationships between samples were evaluated using a PCA approach. As shown in **Fig 1A**, the distribution of the samples is in concordance with the breed histories and in line with previous results obtained for traditional Dutch populations [23]. While, Holstein Friesian occupied the central position, PC1 separated the dual-purpose breeds Meuse-Rhine-Yssel and Deep Red, which are genetically closely related [55], from all others. This is in agreement with the history of these two breeds where Deep Red originated from the Meuse-Rhine-Yssel in the east of the Noord-Brabant province following multiple generations of selection for coat color [55]. The PC2 separated the GWH from other breeds, providing further support for the genetic differentiation of this population. The model-based clustering analysis supported the PCA results. We used the information obtained from the PCA, which showed six different clusters, to run the model-based clustering analysis from *K* = 2 to *K* = 6, and the smallest CV error to estimate the best number of *K* ancestral populations. The results (**Fig 2**) supported the high differentiation of the GWH breed at *K* = 3 in an independent genetic cluster. The separation of Dutch Friesian and Dutch Belted breeds occurred at *K* = 4, and finally the Meuse-Rhine-Yssel and Deep Red formed two distinct clusters at *K* = 5, which had the smallest CV error (0.54), reflecting their close genetic relationship [55]. In this analysis, we included the Holstein Friesian breed, however, determining the extent of admixture in this breed requires further studies of a larger sample size [56]. In the admixture analysis, populations with a low number of samples are less likely to be assigned to their own ancestral cluster and as a consequence, they are depicted across multiple drifted groups [56].

With the separation of the GWH population from the non-white-headed breeds, we decided to investigate if this breed, with ACOP, is also isolated from white headed breeds without pigmentation around the eyes, that are, Hereford and Simmental (without ACOP). The PCA separated the breeds into three clusters based on their genomic information (**Fig 1B**). Animals represented in **Fig 1B** were used for the GWAS in the second step. The PC1, which explains 30% of the observed variation, divided the animals with and without ACOP and confirms the genetic differentiation of the GWH breed. The PC2, divided the Hereford and Simmental breeds into two clear clusters indicating two separate populations in accordance with previous reports [57]. This pattern, which confirms the GWH differentiation was also obtained when the five Dutch breeds and the three commercial populations (Holstein, Simmental, and Hereford) were combined (**S1 Fig**).

### Genomic regions showing significant association with the GWH breed

**Fig 3:**
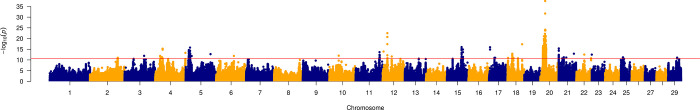
Manhattan plots showing the GWAS results from contrasting GWH animals with the ACOP phenotype and those of the Dutch Belted, Deep Red, Meuse-Rhine-Yssel, Dutch Friesian, and Holstein Friesian breeds without white head and non-ACOP phenotype. The y-axis of the plot represents the -log_10_ (*P*-values) from the GWAS and the x-axis shows the genomic location of each variant. The horizontal red line indicate the significant association (*q-value* ≤0.001) at the genome-wide level.

The GWA study was used to identify and characterize genome regions that differ between GWH and other breeds to find candidate genes possibly associated with pigmentation and/or eye protective phenotypes e.g. ACOP, which is typical of GWH breed. Animals with ACOP (GWH) were classified as cases and animals of the Dutch Belted, Deep Red, Meuse-Rhine-Yssel, Dutch Friesian, and Holstein Friesian breeds were considered as controls. At the genome-wide level (*q-val*< = 0.001), 137 genome hits (**S3 Table** and **Fig 3**) with more than one significantly associated variant were detected. The associated regions were distributed across the 29 chromosomes (**Fig 3**) and the regions with the most significant associations (*p-value* <-4.9E-14) and with the highest number of associated variants (>100 significant associations) mapped to BTA4 (20.0–29.9 Mb and 116.8–118.8 Mb), BTA5 (10.1–19.7 Mb), BTA12 (12.0–18.5 Mb), BTA15 (50.6–59.8 Mb and 60.3–67.8 Mb), BTA20 (10.2–19.9 Mb and 20.2–29.5 Mb) and BTA21 (0.4–8.8 Mb). A total of four genomic regions co-localized with those detected by Pausch et al. [9] in Fleckvieh breed, which are, two regions located on BTA5 (10–19.7 Mb; 57.5–58.9 Mb), one on BTA13 (50.1–59.9 Mb) and one on BTA22 (30.4–32.5 Mb). The low coincidence between the studies may indicate that most associations are breed-specific suggesting that this phenotype may have a different genetic background in these breeds. However, multiple methodological and biological factors can influence these differences. Pausch et al. [9] used genomic information from a combination of SNP arrays (version 1 and 2 of Illumina BovineSNP 50K Bead chip®, and Illumina BovineHD Bead chip® 777k), whereas we used whole-genome sequence variants. Additionally, Pausch et al. [9] used a quantitative trait (a proportion of progeny with ACOP) in the GWAS study while in the current study we used the ACOP traits as a binary phenotype. Finally, while large sample sizes are needed for GWAS of complex traits, the sample size can be dramatically reduced for a case and control analysis in binary phenotypes [58, 59].

#### QTL detection in white headed cattle with and without ACOP

As there were no GWH animals with a completely white head and without ACOP, 50 animals from Hereford and Simmental breeds were selected from the 1000 Bull project [24]. These data were merged with variants from our GWH to carry out a GWAS analysis using a total of 15,751,624 variants to: 1) detect GWAS signals associated with the phenotype variation of GWH breed to find candidate genes related with pigmentation and/or eye protection phenotypes, e.g. ACOP, by contrasting breeds with ACOP (GWH) and without ACOP (Hereford and Simmental) and completely unpigmented area around the eyes; and 2) to reduce the number of candidate genomic regions by retrieving the QTLs overlapping with the GWAS (breeds without white head vs GWH). A total of 187 genomic significant hits with at least two significant SNPs were detected (**S4 Table**), and 100 (53.4%) co-localized with the QTLs identified when the six breeds were included in the analysis (**S4 Table**). This result may suggest that those regions specifically affect the GWH breed and may be associated with its color phenotype. Interestingly, the QTL on BTA5 (region, 10.1–19.7 Mb), was also identified by contrasting GWH vs breeds without white head (BTA5, region 10.0–13.7 Mb). Pausch et al. [9] reported the same QTL earlier at BTA5 (15.6–20.6 Mb, remapped to ARS-UCD1.2 assembly) which explained around 7.9% of the total phenotypic variation of ACOP in the Fleckvieh breed [9].

Our GWAS analyses were limited by the fact that significantly associated genomic regions can be observed due to the different genetic backgrounds between the breeds. This confounding effect should either be eliminated through a better study design (e.g. F2 crosses with another white face breed that does not show ACOP) [60–62] or by reducing the number of false positives using a combined approach in a downstream analysis [17, 63]. For example, the application of complementary methods to investigate whether loci significantly associated were recently selected in the population [16], the description of functions of the genes in candidate regions, and finally the experimental validation. As we did not have animals from the GWH breed without ACOP we decided to investigate if our significantly associated genomic regions were recently selected in our GWH population to detect positional candidate genes functionally associated with pigmentation, eye disease, and/or UV protection.

### Detection of a breed-specific selective sweeps in GWH

**Fig 4:**
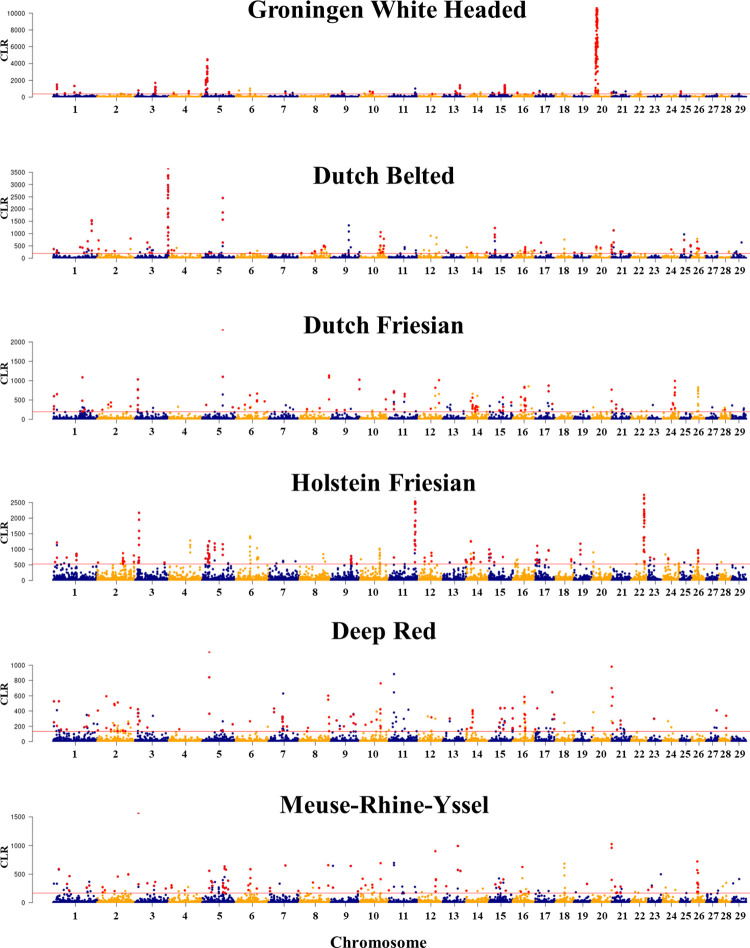
Genome-wide selective sweep scans using SweeD in each breed. Manhattan plots representing the composite likelihood ratio values (y-axis) from SweeD for each marker across the genome (x-axis). The threshold of the significant association (top 1% of the highest composite likelihood ratio values) for declaring candidate selective sweeps is indicated by the red line. Red points indicate candidate genomic regions detected by both the SweeD and OmegaPlus methods.

We used the whole genome resequencing data from six cattle breeds (GWH, Dutch Belted, Deep Red, Meuse-Rhine-Yssel, Dutch Friesian, and Holstein Friesian) to find out breed-specific selective sweeps (BSSS) in the GWH breed with two complementary methods: SweeD, which detects selective sweeps based on the variant frequencies using a composite maximum likelihood approach [39]; and OmegaPlus, that identifies patterns of linkage disequilibrium using the ω statistic [41]. Only significant genome regions (top 1% of the empirical distribution) in both algorithms (SweeD [39] and OmegaPlus [41]) were selected for furher analysis. With this approach, 257 breed-specific putative genomic regions under selection were detected (**Fig 4, S5 Table**). The candidate regions were distributed across the 29 autosomes (**Fig 4**) with sizes that ranged from 3.4 kb to 140.4 kb and a mean of 17.8 kb. The breed with the lowest number of candidate regions was GWH (31), followed by Meuse-Rhine-Yssel (40), Dutch belted (41), Dutch Friesian (46), Holstein Friesian (48), and Deep Red (51). The highly significant BSSS migth indicate “divergence signals” between breeds [3]. Thus, the BSSS might be an indicator of genomic regions affecting unique phenotypic characteristics for which the selection signal was detected [3] and therefore can be used to validate the GWAS signals for the phenotypic variation of the GWH breed. The regions with the most significant associations obtained by both methods were found on BTA5 (12 Mb) and BTA20 (14–20 Mb) in GWH; BTA3 (115–118 Mb) on Dutch Belted and BTA3 (12–13 Mb), BTA11 (93–94 Mb) and BTA22 (45–48 Mb) on Holstein Friesian (**Fig 4**). When we evaluated the co-localization between the BSSS (±500 kb up-and downstream) in GWH and QTLs detected by GWAS, eight genomic regions were also mapped with all methods (**S3–S5 Tables**) as follows: one on BTA4 (70.2–71.3 Mb), one on BTA5 (10.0–19.7 Mb), one on BTA10 (26.9–29.4 Mb), one on BTA13 (60.0–61.4 Mb), one on BTA15 (55.5–59.8 Mb), two on BTA20 (10.0–19.9, 20.0–22.7 Mb) and one on BTA25 (0.5–9.2 Mb).

We also evaluated if the candidate selective sweep co-localized with known bovine QTLs deposited in the AnimalQTLdb [64] database. A total of 4,558 different QTLs affecting 260 traits were found within 257 candidate BSSS (**S6 Table**). Several of the candidate selective sweeps highlighted loci which were mainly associated with milk quality, milk production, feed efficiency, body weight, and several meat-related phenotypes. To be noted, these results are in line with the economic objective established for the studied breeds; Dutch local cattle (Meuse-Rhine-Yssel, Deep Red, and GWH) have been selected for dual-purpose characteristics including milk production. Although our candidate selective sweeps were selected as unique in each breed, we still can find BSSS affecting the same trait. This can be explained by the fact that in livestock populations, including traditional cattle breeds, the selection for economically important traits, e.g. complex traits, might happen across many loci with small effects [2, 65]. The successful identification and characterization of those BSSS that are associated with economically relevant traits can be used to: 1) improve the knowledge about the processes influencing the genetic diversity of each breed; and 2) identify candidate genes and/or causal variants affecting phenotypes under selection. Thus, further studies are encouraged to explore the relationship between our candidate BSSS and the impact that they may have on economically relevant traits in detail as this was not an objective of the current study.

### Several ROH hotspots map to QTLs and putative selective sweeps

**Fig 5:**
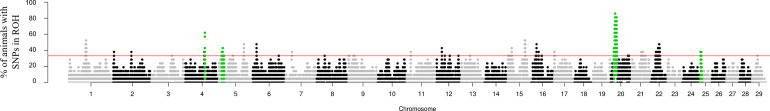
Genome-wide ROH hotspots disribution in GWH breed. The y-axis represents the percentage (%) of animals with SNPs in ROH regions and the x-axis the genomic coordinate of each variant. The significance threshold indicating the genomic regions (ROH hotspots) containing the variants present in more than 99% of a ROH region across the samples is indicated by the red line. Green dots represent genomic regions on BTA4 (70.2–71.3 Mb), BTA5 (10.0–19.7 Mb), BTA20 (10.0–19.9 Mb and 20.0–22.7 Mb), and BTA25 (0.5–9.2 Mb) that co-localize with significant SNPs commonly detected by the GWAS, selective sweeps and ROH hotspots.

The identification of the genomic regions in ROH in the GWH breed was implemented as a complementary method to confirm and reduce the number of candidate genomic regions that co-localize between the GWAS signals and BSSS. We found 4,911 ROH regions that cover on average a total of 207.4 Mb of the genome. Of these ROH regions, around 73% (3,615) can be classified as small (0.5–1 Mb) regions, indicating more ancient consanguinity or population founder effects [66]. This result is common in cattle populations, where longer ROH regions have been found less frequently than shorter ones [67]. To reduce the number of identified genomic regions in ROH, the ROH hotspots were defined by identifying genomic regions containing the variants with the highest frequency (top 1%) in a ROH across the GWH population **(Fig 5**, **S7** and **S8 Tables**). With this approach, 57 genomic regions were detected as ROH hotspots. With their genomic coordinates, we were able to reveal genomic regions that co-localize with the previously detected BSSS and GWAS signals. Five genomic regions that mapped to BTA4 (70.2–71.3 Mb), BTA5 (10.0–19.7 Mb), BTA20 (10.0–19.9 Mb and 20.0–22.7 Mb), and BTA25 (0.5–9.2 Mb) were overlapped between the three methods, and thus genes on those regions are probably under selection in the GWH breed [68, 69].

### Positional and functional candidate genes associated with pigmentation and retinal diseases

We also investigated whether the function of the positional candidate genes are specifically associated with pigmentation and/or metabolism of melanocytes. First, we focused on genes that mapped to regions that overlapped between ROH hotspots, BSSS, and the GWAS signals (GWH vs other Dutch breeds, and GWH vs Hereford and Simmental breeds) (**Fig 5**). These regions included 141 genes (**S9 Table**), of which some are functional candidate genes. For example, on BTA 5 (12–17 Mb), the transmembrane o-mannosyltransferase targeting cadherins 2 (*TMTC2*) located at 12.2 Mb, is associated with calcium ion homeostasis [70]. Calcium homeostasis is of major importance in melanocytes and is suggested to be regulated by melanosomes [71]. The KIT Ligand (*KITLG*) locus (BTA 5, 18.2–18.3 Mb), which encodes a ligand for the receptor-type-tyrosine kinase KIT and contributes to coat color in various species, including cattle [72, 73]. On BTA20 (10.9–20 Mb), the region with the most significant SNPs contains the *DEPDC1B* (DEP domain-containing protein 1B) gene at position 18.5–18.6 Mb, which is associated with the hyperproliferation of abnormal melanocyte cells [74]. This gene is overexpressed in melanoma and encodes DEPDC1B protein that contains a DEP domain [75, 76], which plays an active role in controlling cell functions, including specific signal of retinal photoreceptor and cell polarity [76, 77].

Interestingly, there are two genes (**S7 Table**) in our list related with retinal diseases, for example *CWC27* (CWC27 Spliceosome Associated Cyclophilin) associated with Retinitis Pigmentosa [78]; on BTA25 (1.1–1.2 Mb) the function of the Clusterin Associated Protein 1 (*CLUAP1*) in the vertebrate eye is important for ciliogenesis and photoreceptor maintenance [79]. Although only few cases of eye degenerative diseases with a genetic background have been reported in cattle [80–82], recently Michot et al. [83] evidenced a group of mutations related with eye diseases that are segregating in European cattle breeds with direct impact on animal health e.g., the recessive frameshift mutation on *RP1* gene that causes loss of vision in cattle populations.

### The most significant SNPs on BTA20 mapped to genes related with UV protection and melanocyte differentiation

The analysis of the whole genome resequencing data allowed to identify variants within candidate genomic regions that can help to clarify the cause of the phenotypic differences that exist between GWH and the remaining breeds. We investigated the genomic regions on BTA20 (10.0–19.9 Mb and 20.0–22.7 Mb) because those regions contained the most significant associations at three levels (GWAS, **Fig 3**; BSSS, **Fig 4**; and ROH, **Fig 5**). We studied the top ten significant SNPs in these regions to identify putatively associated genes. Nine of these SNPs mapped to four genes (*RAB3C*, *NDUFAF2*, *ZSWIM6*, and *PDE4D*; **Table 2**), and 11 of them to intergenic regions (**Table 2**). The linkage desequilibrium between those SNPs was high, ranging from r^2^ = 0.91 to one (**Table 2**), and one of these SNPs (rs381052637, *p-value* = 8.64E-22) mapped to the 3′UTR of the *PDE4D* gene. SNPs located in 3′-UTR sequences may abolish or create a microRNA target and consequently may lead to different activities of the gene thereby contributing to interindividual variability [84, 85].

**Table 2:** Genomic localization of the most significant SNPs on BTA20.

GWAS results					Candidate Genes			
BTA	Position (bp)	SNP ID	Localization	*p-value*	BTA	Gene Start	Gene End	Gene Symbol
20	17,974,182	rs382263925	intronic	5.89E-23	20	17,842,584	18,052,859	*ZSWIM6*
	18,330,187	rs381810091	intronic	1.74E-24		18,210,359	18,370,943	*NDUFAF2*
	20,044,595	20:20044595	intronic	8.64E-22		20,014,955	20,315,593	*PDE4D*
	20,044,910	rs380360322	intronic	8.64E-22				
	20,278,747	20:20278747	intronic	8.64E-22				
	20,314,517	rs381052637	3 prime UTR	8.64E-22				
	20,524,538	20:20524538	intronic	8.64E-22		20,440,181	20,735,999	*RAB3C*
	20,542,032	20:20542032	intronic	8.64E-22				
	20,598,559	20:20598559	intronic	8.64E-22				

^1^BTA: *Bos taurus* chromosomes, Position (bp): position in base pair of SNP, SNP ID: Variant displaying the significant association with GWH breed, *p-value*: nominal *p-value*.

*Linkage disequilibrium based on the squared correlation (*r* ^2^) from genotypic allele counts was higher than 0.91 between presented SNPs.

Four of the most significant SNPs (**Table 2**) mapped to the Phosphodiesterase 4D (*PDE4D*) gene. *PDE4D* is involved in the degradation of the Cyclic AMP. In humans, the skin pigment production and its protection against the UV radiation improved with the up-regulation of cAMP in melanocytes [86]. However, the function of PDE isoforms in pigmentation and melanocyte biology has not been extensively studied. Khaled et al. [87] reported that the up-regulation of *PDE4D* loci mediated by the MC1R-cAMP-MITF pathway led to a reduced melanocyte pigmentation in mice [88–90]. Interestingly, genes in the MITF pathway have been linked in many cattle breeds with coat color phenotypes [11, 91, 92], and also in other species [93]. As far as we know, there is no evident relationship between the Ubiquinone Oxidoreductase Complex Assembly Factor 2 (*NDUFAF2*) or Related Protein Rab-3C (*RAB3C*) genes with coat color or melanogenesis. However, the *RAB3C* gene is part of the Rab GTPases proteins, which were involved in cell membrane trafficking and associated with melanosomes [94]. Finally, another interesting candidate gene that maps to 40 kb downstream of the rs381810091 SNP (*p-value* = 1.74E-24) is the ERCC Excision Repair 8 (*ERCC8*) gene, involved in protein ubiquitination and UV response. In humans, the *ERCC8* gene is associated with Ultraviolet-sensitive syndrome [95] a genetic disorder characterized by cutaneous photosensitivity that causes differentiated skin pigmentation and greater freckling, without an increased risk of skin tumors [95, 96].

## Conclusion

The used integrative approach based on the combined use of GWAS, selective sweep and ROH analyses identified several regions of the cattle genome (BTA4,70.2–71.3 Mb; BTA5,10.0–19.7 Mb; BTA20,10.0–19.9 Mb, and 20.0–22.7 Mb; and BTA25,0.5–9.2 Mb) as candidates to explain phenotype variation in the GWH breed. Importantly, those regions contained breed-specific genetic markers and candidate genes that are functionally related with pigmentation (e.g. *PDE4D*), UV protection (e.g. *ERCC8*), or retinal degeneration (e.g. *CWC27*, and *CLUAP1*). This finding contributes to characterizing the genetic background of the GWH breed and provides insights to further investigate the biological pathways and causative mutations influencing skin pigmentation and/or eye protective phenotypes e.g. Ambilateral Circumocular Pigmentation, and the biological implications of skin pigmentation for animal adaptation.

## Supporting information

S1 Fig Principal component analysis of the 170 cattle samples from five local Dutch Breeds (Dutch Belted, Dutch Friesian, Meuse-Rhine-Yssel, Deep Red, and GWH) and three commercial breeds Holstein Friesian, Hereford and Simmental. Individuals from the GWH breed (red circle) were distantly positioned from all other breeds.
(TIF)

S1 Table Read coverage and number of variants by animals and breeds from five local Dutch Breeds (Dutch Belted, Dutch Friesian, Meuse-Rhine-Yssel, Deep Red, and GWH) and the commercial breed Holstein Friesian. (XLSX)

S2 Table Variant effect predicted from whole-genome variant from Dutch Belted, Dutch Friesian, Meuse-Rhine-Yssel, Deep Red, Holstein Friesian, and GWH breeds. (XLSX)

S3 Table Genome-wide significant QTL for GWH and five cattle breeds without white head (Dutch Belted, Deep Red, Meuse-Rhine-Yssel, Dutch Friesian and Holstein Friesian). (XLSX)

S4 Table Genome-wide significant QTL for phenotypic variation of GWH from contrasting Simmental, Hereford white headed breeds. (XLSX)

S5 Table Breed-specific selective sweep regions detected in Dutch Belted, Dutch Friesian, Meuse-Rhine-Yssel, Deep Red, GWH, and Holstein Friesian breeds. (XLSX)

S6 Table Quantitative trait locus, from the Animal QTL database, in breed-specific selective sweeps detected in Dutch Belted, Dutch Friesian, Meuse-Rhine-Yssel, Deep Red, Holstein Friesian and GWH breeds. (XLSX)

S7 Table Genomic distribution of SNPs in RHO hotspots in GWH breed. (XLSX)

S8 Table Genomic coordinates of RHO hotspots in GWH breed. (XLSX)

S9 Table Genes that mapped to genome regions on BTA4 (70.2–71.3 Mb), BTA5 (10.0–19.7 Mb), BTA20 (10.0–19.9 Mb, and 20.0–22.7 Mb) and BTA25 (0.5–9.2 Mb). (XLSX)
