# Supplementary figures and images for: Integrative QTL mapping and selection signatures in Groningen White Headed cattle inferred from whole-genome sequences
Source: PLoS One. 2022 Oct 26;17(10):e0276309. doi: 10.1371/journal.pone.0276309 (PMC9605288; doi:10.1371/journal.pone.0276309)

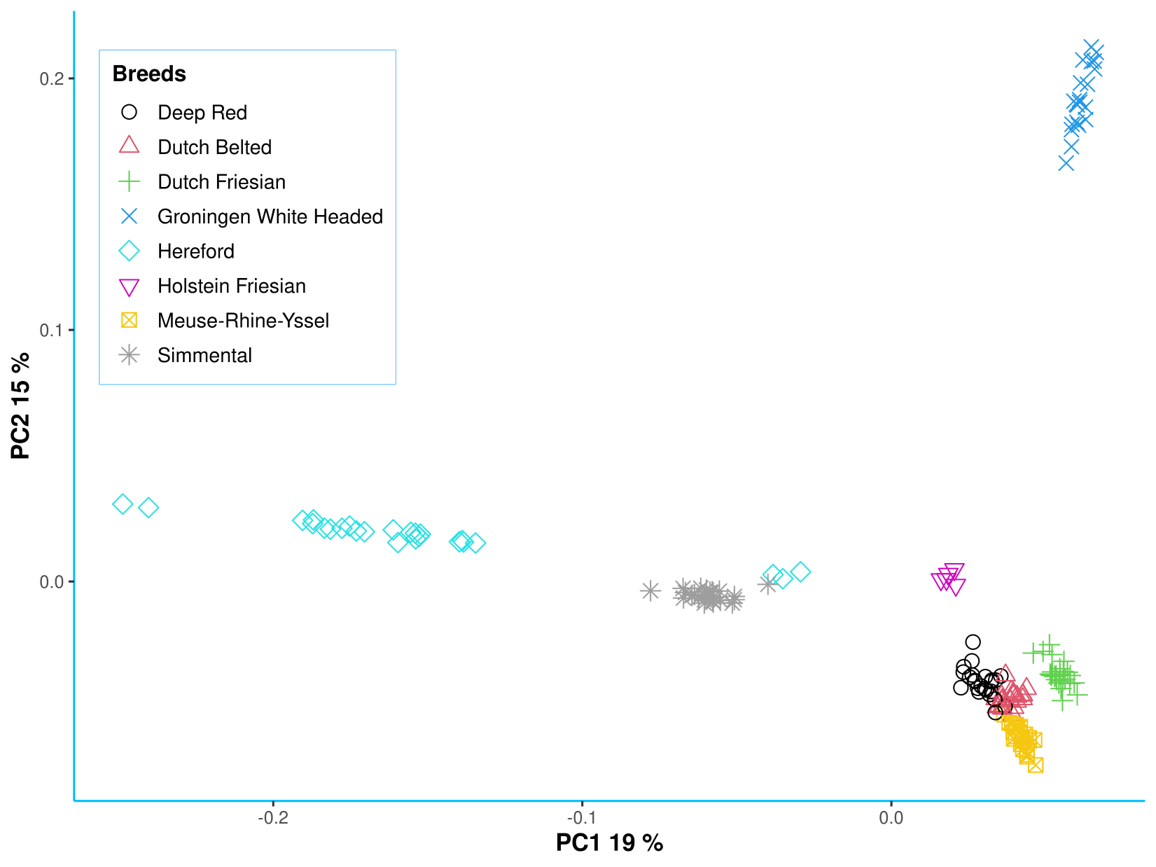

Supplement: S1 Fig — Individuals from the GWH breed (red circle) were distantly positioned from all other breeds. (TIF) [file pone.0276309.s001.tif]
